# Understanding dental caries as a non-communicable disease

**DOI:** 10.1038/s41415-021-3775-4

**Published:** 2021-12-17

**Authors:** Nigel B. Pitts, Svante Twetman, Julian Fisher, Philip D. Marsh

**Affiliations:** 41415106832001 Faculty of Dentistry, Oral & Craniofacial Sciences, King´s College London, Tower Wing, Guy´s Hospital, London, SE1 9RT, UK; 41415106832002 Department of Odontology, Faculty of Health and Medical Sciences, University of Copenhagen, Nørre Alle 20, 2200 Copenhagen N, Denmark; 41415106832003 Department of Oral Diagnostics, Digital Health and Health Services Research, CharitéCentrum 3 für Zahnmedizin, Charité-Universitätsmedizin Berlin, Aβmannshauser Str 4-6, 14197 Berlin, Germany; 41415106832004 Department of Oral Biology, School of Dentistry, University of Leeds, Leeds, LS2 7TF, UK

## Abstract

The recent developments in the science underpinning our knowledge of both the initiation of dental caries and the subsequent behaviour of lesions over time gives us a solid base to understand caries differently. Advances in understanding the human and oral microbiome have come in parallel with the recognition of the importance of balancing protective and pathological risk factors. Caries prevention and management is now about controlling risk factors to maintain a balanced intraoral biofilm ecology that guards against a continuing low pH driven by the frequent consumption of sugars. Thus, caries control is no longer about attempts at eradicating any specific microorganism. Further, the present knowledge leads to the classification of dental caries as a non-communicable disease (NCD), which is vitally important from a policy perspective (both globally and at the country level). Caries shares similar risk factors with other chronic/systemic diseases, which provides opportunities for developing common prevention strategies and promoting health equity through action on the social determinants of health. So, preventing and controlling caries should be integrated across the so-called upstream, midstream and downstream levels and these activities can also help to control other NCDs.

**Supplementary Information:**

Zusatzmaterial online: Zu diesem Beitrag sind unter 10.1038/s41415-021-3775-4 für autorisierte Leser zusätzliche Dateien abrufbar.

## Key points

- Advances in the understanding of the oral microbiome and the caries process call for re-evaluation of caries prevention and management.
- Measures counteracting low pH conditions within the oral biofilm support a balanced and health-associated microbiota.
- Dental caries shares similar risk factors with other non-communicable diseases, and its integrated prevention and management may have a positive impact on overall health.

**Supplementary Information:**

Zusatzmaterial online: Zu diesem Beitrag sind unter 10.1038/s41415-021-3775-4 für autorisierte Leser zusätzliche Dateien abrufbar.

## Introduction

Dental caries is a major health problem in most industrialised countries, in which the majority of children and adults experience the disease.^1^ In the Global Burden of Disease Study,^2^ untreated caries was the most prevalent of all 291 medical conditions evaluated, affecting 3.1 billion people (44%) worldwide,^3^ with a major impact on quality of life and high costs for individuals, families and society. The disease is unevenly distributed in populations with a strong socioeconomic gradient.^4^ Similar with other conditions that are described as non-communicable diseases (NCDs), dental caries develops as the result of a combination of genetic, physiological, environmental and behavioural factors.^5^ A serious concern is that although dental caries is largely a preventable disease, its prevalence has barely reduced over the last 30 years.^6^ This paper argues that recognising dental caries as an NCD rather than an infectious disease will enable caries to be integrated into strategies on oral health promotion, prevention and treatment, and into overall NCD policies.^7^

### The human microbiome

Humans are composed of equal numbers of eukaryotic and microbial cells.^8^ These microorganisms, termed the human microbiome, are natural and colonise all environmentally exposed surfaces of the body, from where they deliver functions essential to our wellbeing. The human microbiome plays a fundamental role in digestion and energy production, the normal development of the host defences and many of our physiological systems.^9^^,^^10^ It also acts as a barrier to colonisation by exogenous and often pathogenic microbes. Generally, we live harmoniously with our microbiome, but on occasions, this relationship can break down and disease can occur. The breakdown is termed dysbiosis and is usually a consequence of a major change to the habitat that disturbs the delicate balance between the microbiome and the host. The imbalance may lead to a range of various conditions along the microbiome-gut-brain axis, such as autoimmune and inflammatory-mediated diseases, malnutrition, obesity and neurological disorders.^9^^,^^11^

The shaping of our resident microbiome starts at birth and continues across the lifespan, but 'acquisition' of bacteria should be contrasted with 'infection'. Infectious diseases are caused by pathogenic microorganisms and can be spread, directly or indirectly, from one person to another (that is, they are 'communicable'). These microorganisms are not generally found at healthy sites, although in certain situations, they can be carried asymptomatically or lie dormant until re-activation occurs when circumstances change (for example, immunity is compromised). Many pathogens produce specific virulence factors, and the detection of such a pathogen can be diagnostic for a particular disease. Approaches to control classical infectious diseases include elimination or exclusion of the pathogen; for example, with antibiotics or vaccines.

#### The oral microbiome

The mouth harbours a complex microbiome that persists and grows on oral surfaces as multi-species biofilms; these biofilms are termed dental plaque when they develop on teeth.^12^ The unique properties of the oral cavity make the composition of the oral microbiome characteristic of the site but distinct from that of neighbouring habitats, such as the skin and the digestive tract. These observations emphasise an important principle, namely the decisive role played by the local environment in determining which species can colonise, grow and become either major or minor components of the microbiome at a specific niche. The oral microbiome has a symbiotic relationship with the host.^12^^,^^13^ The resident oral microbes exhibit pathogen exclusion, downregulate undesirable and potentially pro-inflammatory responses to beneficial resident organisms,^14^ and promote cardiovascular health by the enterosalivary nitrate-nitrite-nitric oxide pathway.^15^ The relationship between the microbiome and the host is dynamic and is susceptible/vulnerable to change if there are substantial changes to the habitat. This includes the social determinants of health that shape the distribution of the four main behavioural risk factors of NCDs: unhealthy diet, physical inactivity, tobacco smoking and excess alcohol consumption. Online Supplementary Table 1 lists some factors that may influence the composition of the oral microbiota over the lifespan and predispose for subsequent oral diseases.

**Table 1:** Differences between dental caries and classical infections and communicable diseases

Infection	Communicable disease	Dental caries
Microbial aetiology is diagnostic of disease	Yes	No
Pathogen is present in health	No	Yes/often
Pathogen satisfies Koch's postulate	Yes	No
Pathogen produces specific virulence factors	Often	No
Disease is transmitted person-to-person	Yes	No

#### The oral microbiome and dental caries

Early culture-based cross-sectional studies found a correlation between mutans streptococci and caries, but these bacteria are also present at caries-free sites and caries could occur in the apparent absence of these bacteria.^16^ Longitudinal trials provide the best evidence for causality, however, since these can detect temporal changes in the microbiota before caries diagnosis. To date, a common observation is that dental biofilms display a diverging microbial composition over time, with distinct differences between caries-active and apparently 'caries-free' children. Studies have confirmed the discriminatory role of *S. mutans*, although these organisms account for only a tiny fraction of the bacterial community.^17^^,^^18^ In addition, new species and phyla, such as *Scardovia wiggsiae*, *Slackia exigua*, *Granulicatella elegans* and *Firmicutes*, are described in children developing dental caries while other commensal bacteria (*Streptococcus mitis*, *S. gordonii* and* S. sanguinis)* appear in the dental biofilm of non-carious tooth surfaces.^19^

#### Drivers of dysbiosis in dental caries

For many decades, dental caries was described as an infectious transmissible disease with *S. mutans* named the 'arch-criminal'.^20^ It was believed that these bacteria were infectious agents and that infants acquired this pathogen from their mothers only after the eruption of primary teeth.^21^ Consequently, clinical strategies to prevent or delay transmission of these organisms were suggested, along with attempts to suppress or even kill mutans streptococci in the oral cavity with topical antibacterial substances and vaccines.^20^ However, the 'one pathogen, one disease' paradigm of dental caries has now been replaced by a holistic concept of a microbial community as the entity of pathogenicity.^22^ Studies of people of different ages and with different diets from around the world have demonstrated substantial differences in the composition of the microbiota in biofilms overlying caries lesions, with an enrichment of species with an acid-producing and acid-tolerating phenotype.^23^ Thus, the development of a caries lesion is associated with a shift in the balance of the resident dental microbiota, so that normally minor components of the biofilm become more prevalent. The main driver of such dysbiotic shift is frequent consumption of sugars. The inevitable low pH generated from their metabolism is driving the selection of acid-producing and acid-loving microorganisms at the expense of the beneficial oral bacteria that prefer a pH around neutrality. Similarly, a reduction in the flow of saliva and non-daily mechanical disruption (tooth cleaning) of the dental biofilm will drive similar shifts. Dental caries, therefore, has been described as a microbial 'ecological catastrophe';^24^ implicit in this concept and in the 'ecological plaque hypothesis' is that interference of the drivers of dysbiosis is needed to prevent or control disease.^16^^,^^25^ In this way, dental caries is not an example of a classical infectious or communicable disease (Table 1). An appreciation and acceptance of this concept will have implications for dental practice and public health.

## NCDs - what are they and why are they important?

Prevention and control of caries as a NCD will require coordinated action at national, community and clinical levels. On the global/national level, oral diseases have been identified in the United Nations Political Declaration on the Prevention and Control of Non-communicable Diseases as posing 'a major health burden for many countries and that these diseases share common risk factors and can benefit from common responses to non-communicable diseases'.^26^ The World Health Organisation (WHO) subsequently published the WHO global action plan for the prevention and control of non-communicable diseases 2013-2020.^27^ This included two objectives: 1) 'to reduce modifiable risk factors for NCDs and underlying social determinants through creation of health-promoting environments'; and 2) 'Health systems and universal health coverage: to strengthen and orient health systems to address the prevention and control of NCDs and the underlying social determinants through people-centred primary health care and universal health coverage'. The 2021 WHO Resolution on Oral Health,^28^ approved by the World Health Assembly in May 2021, reinforces these objectives in relation to oral diseases and dental caries. It urges countries to reorient the traditional curative approach and move towards a 'preventive promotional approach with risk identification for timely, comprehensive and inclusive care, taking into account all stakeholders in contributing to the improvement of the oral health of the population with a positive impact on overall health'.^28^ Furthermore, the WHO resolution stresses environmentally friendly and less invasive dentistry that could support countries with their implementation of the Minamata Convention on Mercury, including supporting preventive programmes and setting national objectives for caries prevention and health promotion. This should be based on and aligned with our knowledge and understanding that bacteria play a crucial role in oral as well as general health.

A prerequisite for any community action is that the caries prevalence among children and adults is monitored through regular epidemiological research with quality-assured sampling, training, calibration and reporting. On a community level, caries prevention and health promotion strategies and interventions should support interdisciplinary and integrated people-centred health services. This creates opportunities for transdisciplinary collaboration on oral health literacy. Adults and children should be educated in making healthy lifestyle choices through awareness campaigns in the workplace, as well as school-based activities. The focus should be on healthy eating, physical activity, fluoride exposure, regular oral hygiene and anti-tobacco use according to the common risk factor approach.^29^ There is evidence to suggest that school-based education programmes are sustainable and effective in reducing the consumption of sugar-sweetened beverages when follow-up modules are included.^30^ It is important that caries prevention and training supporting oral health communication is integrated into the training of other health professionals, community health workers and the social care workforce, as well as home-based carers. For example, paediatrician's dietary recommendations to prevent obesity among children are almost identical to those of dental professionals for preventing caries and should therefore be expressed and communicated in a harmonised way.

## Implications for the future of practice and policy and patient care

Caries prevention and health promotion have traditionally relied on fluoride exposure, diet control, thorough oral hygiene and antibacterial measures. Acknowledging dental caries as a NCD certainly does not disqualify these measures but places them into a broader context. Table 2 shows some examples of how various strategies can alter the microbial profile and affect the balance of the oral biofilm. An important step is that oral health professionals must adopt and implement the concept of a balanced microbiome as the basis for caries prevention and that maintaining or restoring symbiosis is the outcome over the life course. This will establish a consistent, coherent and mutually reinforcing foundation for communicating with patients and the public. As for most NCDs, the wider challenge is to create health-promoting environments for communities, families and patients that promote health equity by addressing the social determinants of health. An additional and persistent challenge for oral health professionals is conveying the value of oral hygiene routines for the quality of life. To date, the best evidence comes from motivational interviews and horizontal one-to-one counselling.^31^^,^^32^ The dietary advice should focus on limiting the intake of free sugars and fruit juices. Here, the updated WHO sugar recommendations for children and adults are helpful.^33^ To prevent both dental caries and obesity, there is a strong recommendation that the intake of free sugars should not exceed 10% of the total daily energy intake, which corresponds to less than 50 grams per day. A conditional recommendation is to limit the intake to below 5%. Subjects with this low sugar consumption have fewer caries-related species in their saliva and supragingival plaque than those consuming more sugar.^34^ Free sugars are all kinds of sugar, added by the producer during the preparation of food and by the consumer while eating. Notably, several 'natural' products such as honey, syrup and fruit juices are *de facto* free sugars. Oral hygiene instructions should focus on regular gentle disruption of the biofilm rather than on meticulous eradication. The presence of fluoride in the biofilm around the clock plays a pivotal role in biofilm control. Fluoride can reduce the biofilm sugar stress by lowering the critical pH for enamel dissolution, thereby limiting demineralisation. In addition, fluoride can inhibit many traits associated with dental caries, including enzymes associated with biofilm matrix production and enolase, thereby directly slowing down glycolysis while indirectly reducing bacterial sugar transport systems.^35^ Inhibition of acid production removes the environmental conditions that are both responsible for the suppression of beneficial oral bacteria and essential for the enrichment of acid-tolerating species.^36^ A recent study demonstrated that fluoride exposure induced a dramatic shutdown of sugar metabolism in oral biofilms with a subsequent reduction of saccharolytic organisms, underpinning the beneficial effect of fluoride-containing oral hygiene products.^37^

**Table 2:** Potential ways in which caries preventive strategies can possibly contribute to maintain or restore a balanced microbiome

Measure	Mechanism	Effect on pH/biofilm composition
Sugar reduction (amount/frequency)	Less substrate available for acid production and biofilm matrix	Aciduric bacteria less favoured
Fluoride	Decrease metabolic activity in biofilm	Less acid production/inhibition of bacterial metabolism linked to caries
Daily tooth brushing	Regular gentle biofilm disruption, restricts biofilm accumulation	Control growth and climax communities
Saliva stimulation	Enzymes and protective agents, delivers innate host defences	Stabilises the oral biofilm
Prebiotics and probiotics	Favours growth of beneficial oral bacteria	Restricts growth of caries-associated species; less acid production

In addition to these 'traditional' measures, alkali-generating pathways, as well as prebiotic and probiotic strategies, are suggested for the maintenance of a stable and symbiotic oral microbiome. Among the prebiotic agents, xylitol and arginine display well-proven anti-caries properties from clinical trials.^38^^,^^39^ Confectionery and drinks with alternative sweeteners that cannot be metabolised to acid by oral bacteria, but which stimulate saliva, can play a role in preventing a dysbiotic biofilm stress. The use of probiotic bacteria can support biofilm diversity, influence the composition of the oral biofilm and increase the intraoral pH.^40^^,^^41^ In particular, early-in-life exposure to probiotic bacteria may support the acquisition of beneficial strains and possibly decrease the risk for early childhood caries.^42^ Recently, strains have been isolated from caries-free individuals that are both arginolytic and inhibitory to the growth of mutans streptococci.^43^^,^^44^ One of these strains, *S. dentisani*, improved clinical and microbiological parameters associated with oral health in a clinical trial, thereby supporting its use to prevent tooth decay.^41^

## Conclusions

Dental caries is a consequence of a deleterious shift in the composition of dental biofilms to a microbial community dominated by an acid-tolerant and acid-producing microbiota with reduced levels of beneficial bacteria. The shift is driven by similar modifiable risk factors and social determinants as all major NCDs, particularly a poor diet with a high content of free sugars. Our analysis of the evidence leads us to conclude that dental caries is an example of a NCD. Caries prevention should therefore be a part of the chronic disease management approach to tackle the general burden of NCDs,^45^ with special emphasis on disadvantaged groups to reduce inequalities in oral health. Future preventive technologies in practice should reduce the extent and frequency of periods of low pH in the dental biofilm and maintain the pH around neutrality in order to support health-associated communities of beneficial oral bacteria.

## Summary for health professionals

We have presented data to support the concept that dental caries is a preventable NCD. As such, caries shares risk factors with other NCDs and the prevention and management should therefore rely on a similar model of chronic disease management. Excessive sugar consumption is the most important single factor, driving the oral microbiome from a healthy symbiotic balance with the host to a state of dysbiotic imbalance. The goal of primary prevention is to preserve and maintain symbiosis in the oral biofilm while the goal of secondary prevention is to restore an unhealthy microbiome by removing the drivers of these adverse changes. The pillars of primary caries prevention in all age groups are sugar reduction and daily tooth brushing with fluoride toothpaste. Regular exposure to fluoride from the eruption of the first tooth builds on strong evidence from randomised controlled trials, while support for sugar reduction and tooth cleaning derive from prospective cohort studies. The early start of a healthy lifestyle is essential, and dietary guidelines for caries prevention are identical to those for preventing obesity. It is therefore desirable to integrate caries prevention with general wellbeing and vaccination programmes when children and parents meet non-oral health professionals. Parents should be encouraged not to add any free sugars to food and drinks before the age of two years, subsequently following the WHO sugar recommendations for children and adults; free sugars should constitute less than 10% of the daily energy intake to prevent dental caries and obesity. Parents should brush their children's teeth twice daily with an amount of standard fluoride toothpaste adjusted to the age of the child. We recommend that children with weak parental support and poor compliance are referred to oral health professionals. Parent coaching and cultural-specific competence for motivational interviewing may increase this compliance. Professional applications of high-fluoride varnishes are effective for primary and secondary caries prevention. In deprived residential areas, preschools and school settings display an excellent arena for community-based oral health programmes. School staff and family supportive structures, such as nutritionists, speech therapists and social workers, may offer an extended skill mix in communication and behaviour modification. Root caries is a growing health problem among the elderly due to exposed root surfaces, impaired salivary function, polypharmacy and loss of motor skills. The need for 'quick energy' among senior citizens can fuel the caries process and many elderly people, particularly those with diminishing autonomy, require assistance in carrying out their daily oral hygiene and remembering their fluoride products. Subjects of all ages with malignant and severe chronic diseases and/or suffering from dry mouth must consult oral health professionals for an individual care plan.

## Supplementary Information

Supplementary Table 1 (DOCX 14KB)
