# Supplementary material for: Understanding dental caries as a non-communicable disease
Source: Br Dent J. 2021 Dec 17;231(12):749–53. doi: 10.1038/s41415-021-3775-4 (PMC8683371; doi:10.1038/s41415-021-3775-4)
Supplement: Supplementary file 1 — Supplementary Table 1 (DOCX 14KB) [file 41415_2021_3775_MOESM1_ESM.docx]

**Supplementary Table S1.** Factors from the cradle to the grave that may affect and/or disrupt a healthy oral microbiome, with subsequent long-term consequences for oral and systemic health.

| Perinatal factors |  | Genetics |
| --- | --- | --- |
|  |  | Mode of delivery, caesarean section |
|  |  | Early transmission and acquisition of maternal bacteria |
|  |  | Breastfeeding, pacifiers |
|  |  | Treatment with antibiotics early in life |
|  |  |  |
| Lifestyle factors |  | Eating habits, intake of sucrose (amount and frequency) |
|  |  | Drinking habits, especially sweet and carbonated drinks |
|  |  | Sexual behaviour |
|  |  | Smoking, snuffing, drug addiction |
|  |  |  |
| Physiological ageing and drugs |  | Reduced saliva secretion rate and altered composition |
|  |  | Xerogenic drugs, sugar-containing drugs, polypharmacy |
|  |  | Cognitive impairment and lost skills for oral hygiene |
|  |  |  |
